# Crack Healing and Mechanical Properties Recovery in SA 508–3 Steel

**DOI:** 10.3390/ma12060890

**Published:** 2019-03-17

**Authors:** Yao Qiu, Ruishan Xin, Jianbin Luo, Qingxian Ma

**Affiliations:** 1 Department of Mechanical Engineering, Tsinghua University, Beijing 100084, China; qiuy15@mails.tsinghua.edu.cn (Y.Q.); ruishanxin@163.com (R.X.); ljblqw@mail.tsinghua.edu.cn (J.L.); 2 Key Laboratory for Advanced Materials Processing Technology of Ministry of Education, Tsinghua University, Beijing 100084, China; 3 HBIS Group Technology Research Institute, Shijiazhuang 050023, China

**Keywords:** internal crack healing, mechanical properties recovery, tensile properties, impact properties, low cycle fatigue properties, fracture morphology

## Abstract

Internal cracks could be healed under the process of hot plastic deformation. In this study, mechanical properties recovery after crack healing in SA 508–3 steel were investigated. Microstructures of the crack healing zones were observed using an optical microscope (OM) and electron back scattered diffraction (EBSD) technology, and the recovery degrees of mechanical properties in the crack healing zones with the healing temperature and a reduction ratio were tested systematically. The results showed that the internal cracks in SA 508–3 steel disappeared and were replaced by newly formed grains, achieved by recrystallization and abnormal grain growth. The tensile properties of crack healing zones could be fully restored, while their impact and low cycle fatigue properties could only be partially achieved. The recovery degrees of mechanical properties in crack healing zones increased with increasing the healing temperature and reduction ratio in the temperature range of 950–1050 °C. When the temperature was above 1150 °C, the impact properties began to deteriorate because of grain coarsening and larger MA (martensite–austenite) constituents. The microstructural evolution of the crack zone in the SA 508–3 steel was sketched.

## 1. Introduction

During the manufacturing process of heavy forgings, cavity-type defects, such as cracks and voids, are inevitable. Even small-sized defects could aggregate and provoke catastrophic failures. Thus, for the sake of serviceability and economic benefits, the strategy that internal crack in heavy forgings could be healed has attracted much attention.

Griffith [1] first proposed that the process of crack initiation was invertible, but when the crack was small enough, the internal crack could be healed under the mechanism of thermal diffusion. Compared to extensive studies on crack initiation and propagation in metallic materials performed during the last decades, there are few reports on crack healing in metals. Based on the existing researches on crack healing in metallic materials, the healing methods mainly include electro pulsing treatment [2,3,4,5], heat treatment [6,7,8,9], and hot plastic deformation [10,11,12]. From the previous research, the cracks with a width less than 50 µm and a length less than 150 µm could be healed by different healing methods. However, considering the actual production requirements and economic efficiency, hot plastic deformation is the most efficient and most feasible method in the crack healing of heavy forgings. 

Yu et al. [12] systematically analyzed the effects of different process parameters on crack healing in low-carbon steel under hot plastic deformation and found that there is a positive correlation between the recovery degree of the internal crack and heating temperature, holding time, as well as reduction ratio and a negative correlation with the deformation passes and strain rate. Zhang et al. [13] studied the microstructure and hardness of 20 steel with artificially prepared internal cracks. The ferrite grains assembled along the boundary of the internal crack and sub-grains resulted in a higher hardness in the healing ranges than the matrix. Han et al. [14] concluded the regular pattern of inner crack recovery at high temperature based on the physical modelling in 20MnMo steel and pointed out that the diffusion and migration of metal atoms were the mechanism of recovery process. Wei et al. [15] conducted a quasi in situ observation in 1045 steel specimens with real internal cracks and found that the crack healing at crack tips could be achieved after a heat treatment at 1100 °C for 120 min. Zhao et al. [16] also studied the effect of hot isostatic pressing on crack characterization in a laser solid formed Rene888DT superalloy and pointed out that the short cracks can be healed by hot isostatic pressing (HIP) diffusion bonding, while the discrete MC type carbides which precipitated along the healed crack impeded the long cracks healing. Xin et al. [17] systematically investigated the effect of parameters on internal crack healing and pointed out that the recovery degree of crack healing increased with increasing healing temperature, reduction ratio, and holding time and with decreasing strain rate. Xie et al. [18] discovered during the hot compression bonding and post-holding treatment the dissolution and evolution of the interfacial oxides in the bonding joint contributed to the mechanical property recovery of the bonding joint. Recently, a molecular dynamics model was also used to investigate the crack healing mechanism in metallic materials [19,20].

Until now, the existing studies on crack healing in metallic materials mainly focused on microstructure evolution and crack healing mechanism using experimental or numerical simulation methods. Meanwhile, investigations on mechanical properties recovered after crack healing are seldom reported, especially on the impact and fatigue properties recover. Recently, Xin et al. [21] discovered the phenomena that when internal microcracks were eliminated completely under heat treatment healing technique, the tensile properties of the crack healing zones could recover completely, but their impact toughness could only partially be restored. However, most experiments were conducted in the laboratory environment, such as Gleeble simulation. The results obtained from physical modeling experiments laid the foundation of the theoretical description and research, but they could not completely reflect the actual production condition because of the small scale of the samples. In this study, the butt-joint method was used to preset an internal crack in large-sized specimens in order to simulate the real environment condition. A series of experiments on crack healing were carried out during hot plastic deformation and heat treatment, and the mechanical properties of the internal crack healing zones in SA 508–3 steel, including tensile and impact properties, were systematically investigated. Meanwhile, the low-cycle fatigue properties of SA 508–3 specimens with the best percentage recovery of the impact property were studied.

## 2. Materials and Methods 

**Figure 1:**
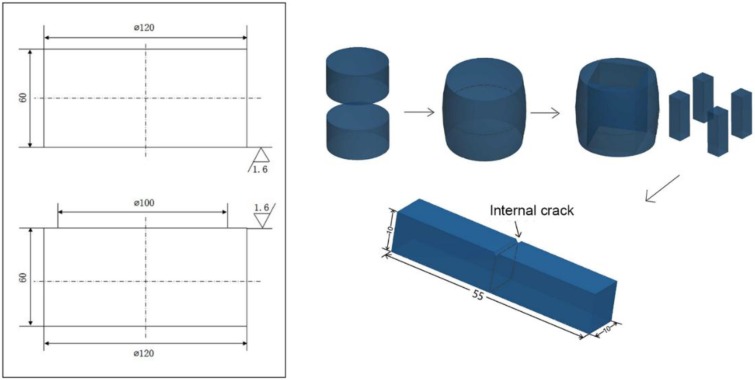
A schematic illustration of the butt-joint method.

The material used in the research was SA 508–3 steel, having a composition (in wt %) of 0.19 C, 0.22 Si, 1.4 Mn, 0.006 P, 0.006 S, 0.12 Cr, 0.53 Mo, and 0.65 Ni. SA 508–3 steel is often used in nuclear power equipment. The samples with internal pre-cracks were prepared via a butt-joint method. The detailed process of crack presetting is shown in Figure 1.

Firstly, SA 508–3 steel was machined into cylindrical samples with a 120 mm diameter and a 60 mm height. Then, to facilitate the subsequent welding, the contact surfaces of the samples were polished into a mirror plane with the roughness of 1.6 µm using a buffing machine, and the edge was chamfered. After cleaning the contact surface, two samples were welded. The plate-shape pre-crack was obtained in the middle part.

To conduct crack healing experiments by the hot plastic deformation method, the samples with internal pre-cracks were heated using an SRX-30–13 batch-type furnace and compressed using an 8MN press machine. 

To study the effect of the reduction ratio and healing temperature, the samples with internal pre-cracks were heated at a heating rate of 100 °C/h from 800 °C to the healing temperatures (850 °C, 950 °C, 1050 °C, 1150 °C, and 1200 °C), and then, the samples were compressed under different reduction ratios (10%, 20%, and 30%) with the strain rate of 0.1 s^−1^. After compression, the samples were held under the healing temperature for 10 h in the furnace, before cooling in the air.

**Figure 2:**
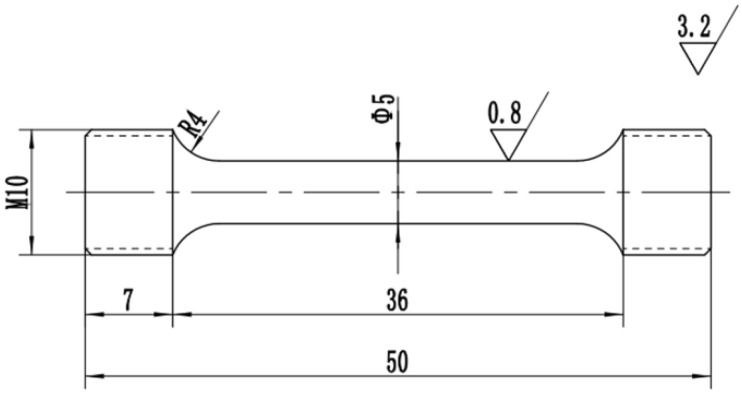
A schematic illustration of the tensile specimens.

Room-temperature tensile tests were conducted to examine the tensile properties recovery of SA 508–3 specimens with crack healing zones by an electronic universal testing machine (model WDW-100). The dimensions of tensile specimens were illustrated in Figure 2. The crack healing zones were confirmed to lie in the middlemost part of the parallel section in the tensile specimens.

The room-temperature pendulum impact tests were performed using a 300 J pendulum impact testing machine. Standard Charpy U-notch specimens were cut from the center of the drum samples with dimensions of 10 mm × 10 mm × 55 mm. The crack healing zones were also confirmed to lie in the middlemost part of the U-notch specimens. The microstructure of the healing zone etched by a picric acid solution was observed by an OLYMPUS-BX51 optical microscope (OM). The EBSD (electron back scattered diffraction) scans with a step size of 0.5 µm were performed using the FIB-710 system, and the analysis data was interpreted by the OIM analysis software. The fractographs were examined using a JSM-7100F scanning electron microscope (SEM).

## 3. Results

### 3.1. Microstructural Morphology of Pre-Crack Zones

**Figure 3:**
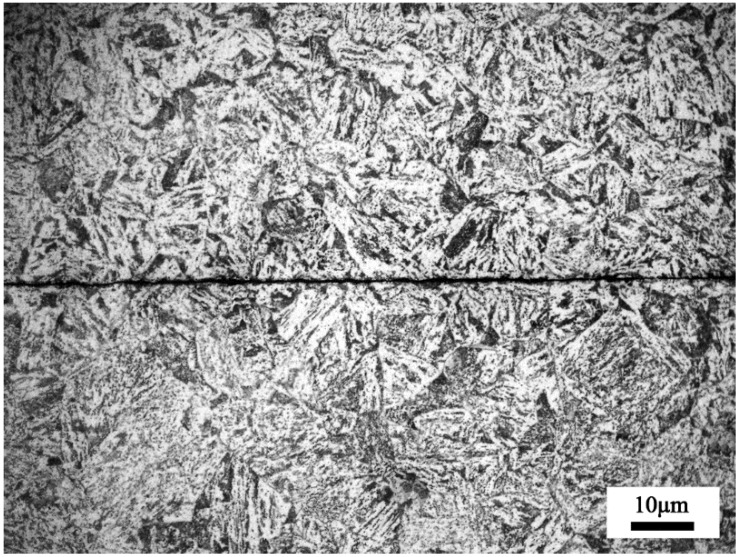
An optical micrograph of the pre-crack zone before the healing treatment.

Figure 3 shows the morphology of the original pre-crack zone in SA 508–3 steel. The pre-crack width of the original internal crack was about 1.4 µm in the middle part of the crack, and the length of the crack was more than 100 mm. The original grain size was 17.24 µm. The original matrix phase was characteristic by lath bainite and martensite.

**Figure 4:**
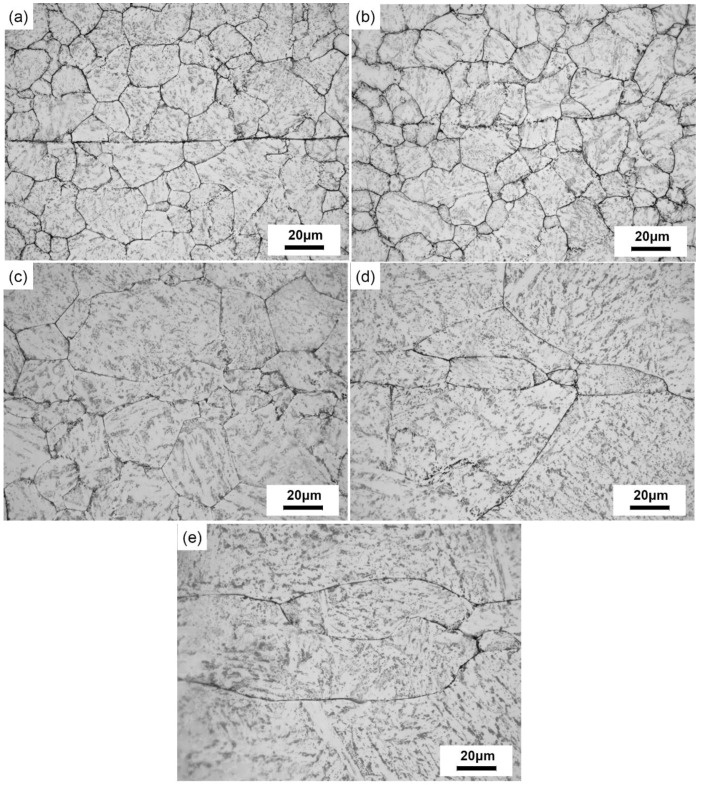
The microstructures on the longitudinal section of the crack healing zones in SA 508–3 steel samples with the reduction ratio of 20% under different healing temperatures for 10 h: (**a**) 850 °C, (**b**) 950 °C, (**c**) 1050 °C, (**d**) 1150 °C, and (**e**) 1200 °C.

Figure 4 shows the microstructural morphology of crack zones under different healing temperatures.

**Figure 5:**
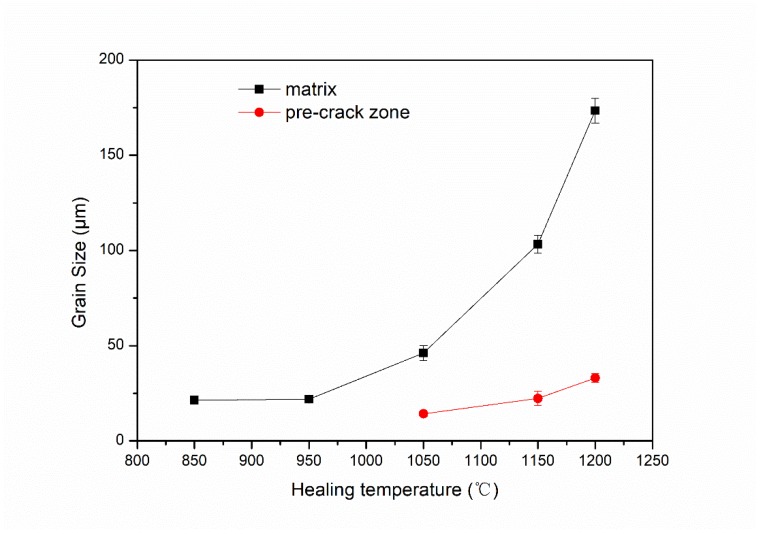
The grain sizes of the matrix and pre-crack healing zones in SA 508–3 steel samples under different healing temperatures.

When the healing temperature was 850 °C (Figure 4a), the crack width was smaller than the original pre-crack. When the temperature was raised to 950 °C (Figure 4b), the crack was segmented into different small parts. While at 1050 °C (Figure 4c), the pre-crack almost disappeared and small-sized grains were observed in the original crack zone, indicating that the internal crack was fully healed. Meanwhile, the grains of the matrix grew rapidly, and there were distinct differences between the microstructures of the crack zone and the matrix. When the healing temperature was above 1150 °C (Figure 4d,e), the microstructures of crack healing zone consisted of coarse grains. The average grain sizes of the matrix and pre-crack zone in different temperatures were shown in Figure 5.

When the sample was healed at 1150 °C, recrystallization was the primary crack healing mechanism [21]. The growth of the recrystallized grains is usually described as normal grain growth. However, after holding at the 1150 °C for 10 h, newly formed grains in the crack healing zones had a preferential orientation, in other words, abnormal grain growth, resulting in elongated large grains that can be observed in the present study.

**Figure 6:**
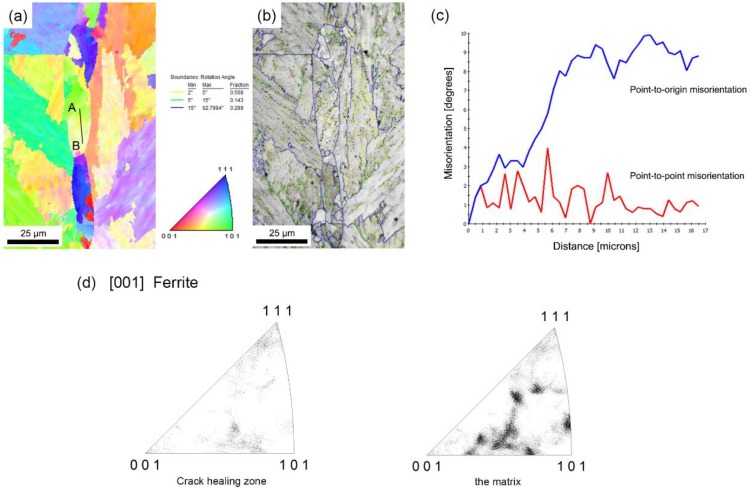
(**a**) An inverse pole figure (IPF) map of the healing zone healed at 1150 °C, (**b**) the corresponding grain boundary map, (**c**) the misorientation profile along the line AB, and (**d**) the IPFs on the crack healing zone and the matrix.

The inverse pole figure (IPF) map of the SA 508–3 steel sample healed at 1150 °C is shown in Figure 6a, and the corresponding grain boundary character distribution map is shown in Figure 6b. Only granular bainite grains were found in the crack healing zone and the matrix (Figure 6a), and their orientations were almost similar (Figure 6d). The distribution of the misorientation profile along the line AB in the newly formed grain is shown in Figure 6c. The morphology feature of the grain can be defined by a minor color variation. The point-to-point misorientation does not exceed 4°, and the point to origin misorientation increased continuously, which indicates the strain gradient within the orientation of the grain. For the present sample, the abnormal growth may be the result of the presence of the pinning effect rather than the texture effect.

**Figure 7:**
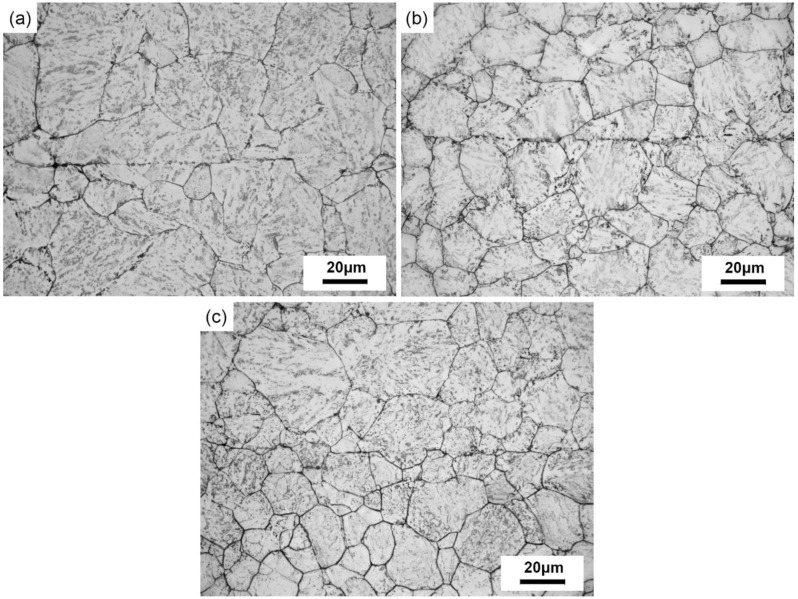
The microstructure of the SA 508–3 after compression at 950 °C with different reduction ratios: (**a**) 10%, (**b**) 20%, and (**c**) 30%.

Figure 7 shows the microstructures of crack zones after compression at 950 °C under various reduction ratios.

The grain sizes and the number of micro-voids decreased with increasing reduction ratio. When the reduction ratio increased to 30% (Figure 7c), the micro-voids and micro-cracks in the crack healing zones disappeared totally and were replaced by newly formed fine grains with a continuous grain boundary.

### 3.2. Tensile Properties Recovery

The tensile properties of SA 508–3 at room temperature should meet the following requirements: the ultimate tensile strength is 550 MPa~700 MPa, the elongation is more than 18%, and the reduction of area is more than 38%.

**Figure 8:**
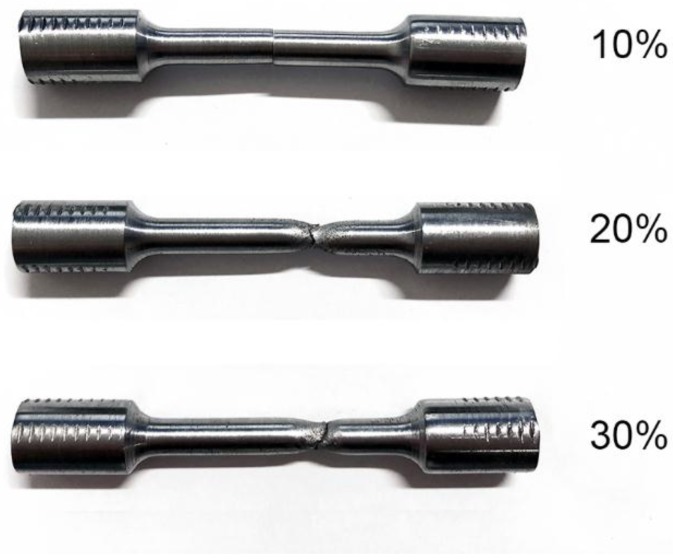
Room-temperature tensile macroscopic fractographs of the crack healing zones under different reduction ratios, healing at 950 °C for 10 h.

Figure 8 shows the room-temperature tensile macroscopic fractographs under different reduction ratios, healing at 950 °C for 10 h.

When the reduction ratio was 10%, there was no necking in the tensile test. When the reduction ratios were above 20%, the apparent extension and displacement occurred on the other two tensile samples. The tensile fracture positions were located in the matrix rather than in the crack healing zone, which indicated the tensile strength of the crack healing zone was higher than the matrix.

**Figure 9:**
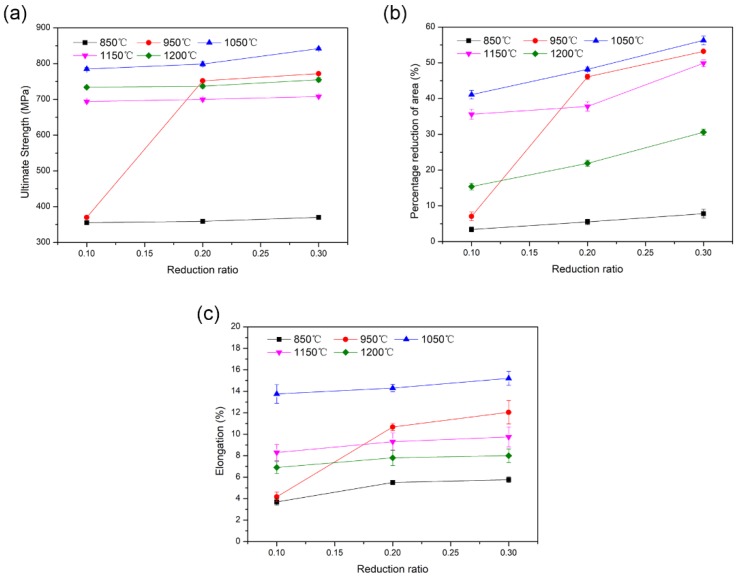
Room-temperature tensile properties recovery of crack healing zones at different healing conditions: (**a**) ultimate tensile strength, (**b**) percentage reduction of area, and (**c**) elongation.

The effect of the healing temperature and reduction ratio on the tensile properties recovery of the crack healing zones are shown in Figure 9.

It was found that the ultimate tensile strength has been recovered completely at 950 °C, with a reduction ratio of 20% (Figure 9a), while the elongation was still less than 18% when the temperature was 1050 °C and the reduction ratio was 30% (Figure 9c), which indicated that the recovery rate of the tensile strength in the internal crack zone was higher than that of the plasticity recovery.

As shown in Figure 9a,b, the recovery rate of the tensile strength and the elongation of the crack healing zones was basically consistent with the increase of the reduction ratio, at the same healing temperature.

With the same reduction ratio, when the healing temperatures increased from 850 °C to 1050 °C, the ultimate tensile strength and the elongation of the crack healing zones increased significantly. The corresponding microstructural morphology of the healing zones presented the obvious disappearance of internal cracks and the generation of recrystallized grains. However, when the healing temperature was higher than 1150 °C, grain coarsening deteriorated the tensile properties.

**Figure 10:**
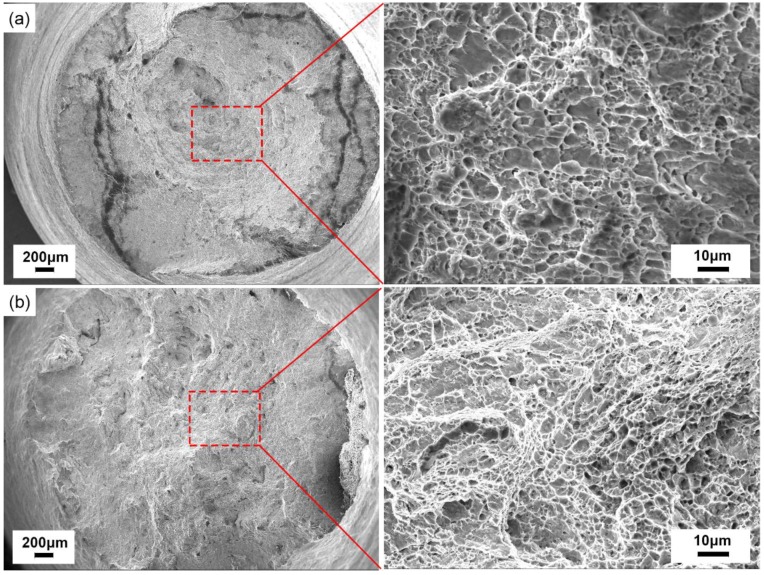
The tensile fracture morphology of the SA 508–3 samples (**a**) at 950 °C with a 30% reduction ratio holding for 10 h and (**b**) at 1150 °C with a 30% reduction ratio holding for 10 h.

The corresponding tensile fracture morphology of the healing zones under different conditions is shown in Figure 10.

With the 30% reduction ratio, the fracture morphology was predominantly a typical equiaxial dimple pattern, as shown in Figure 10a,b. When the healing temperature increased from 950 °C to 1150 °C, the area of equiaxial dimples increased gradually and the diameters of the dimples were smaller. However, the ultimate tensile strength and elongation of the sample healed at 1150 °C were relatively lower. The reason was that lower energy was needed in the micro-void formation during the process of dimple fracture after the sample was healed at a higher healing temperature.

### 3.3. Charpy Impact Properties Recovery

Table 1 gives the room-temperature Charpy impact testing results of crack healing zones under different healing conditions. The selected fracture surfaces were investigated with scanning electron microscopy.

The impact absorbing energy of the SA 508–3 steel original matrix material was tested, and its value was about 47.5 J. The percentage recovery of impact toughness (η) was quantitatively evaluated by the specific value of impact absorbing energy of preset crack samples and the matrix samples. The expression of percentage recovery of impact toughness was defined:

(1) $$\eta =\frac{A_{KU}}{A_{KU0}}\times 100\%$$

where η was the percentage recovery of impact toughness, $A_{KU}$ was the impact absorbing energy after different healing treatment, and $A_{KU0}$ was impact absorbing energy of the original matrix material.

Under our preset crack conditions, the maximum percentage recovery (η) of the impact toughness of internal crack healing samples was about 57% after being compressed 30% at 1050 °C and held for 10 h.

**Figure 11:**
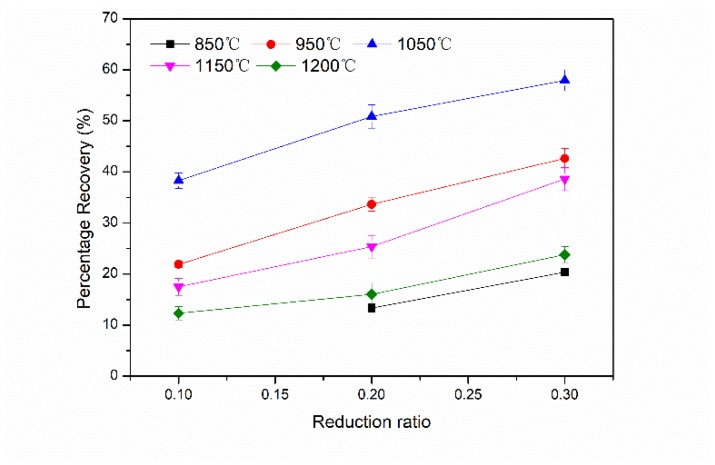
The percentage recovery of room-temperature Charpy-absorbed energy under various healing conditions.

Figure 11 exhibits the percentage recovery of room-temperature Charpy-absorbed energy under various healing conditions. The percentage recovery of impact absorbed energy of crack healing zone increased with an increasing healing temperature. However, when the healing temperature exceeded 1050 °C, the oversized grains in matrix led to a decrement of the recovery rate. Abnormal grain growth was observed after long-time heat preservation, which deteriorated the impact property. Additionally, with the increase of the reduction ratio, their percentage recoveries increased gradually.

**Figure 12:**
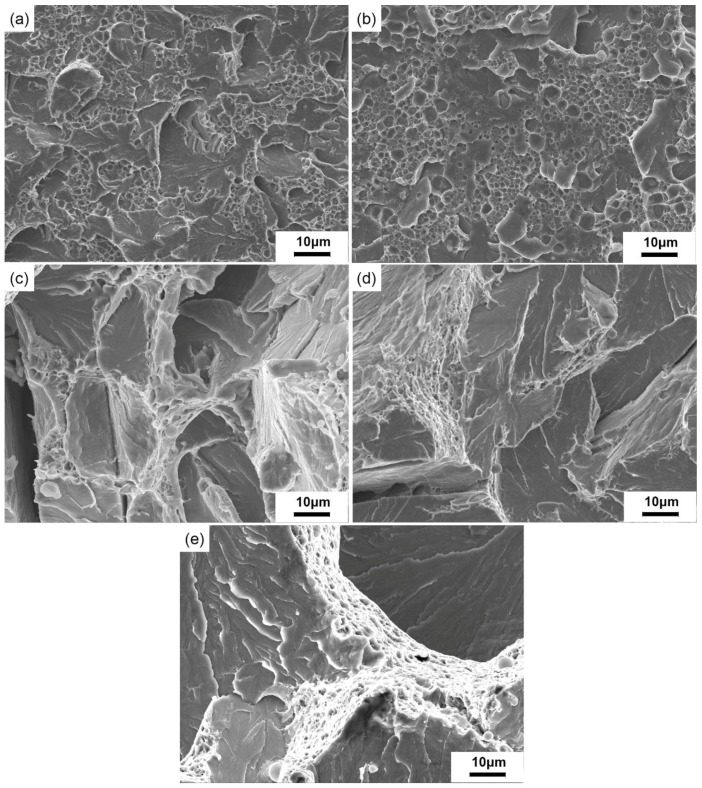
The SEM impact fracture of SA 508–3 under different temperatures when the reduction ratio was 20% (**a**) at 850 °C, (**b**) at 950 °C, (**c**) at 1050 °C, (**d**) at 1150 °C, and (**e**) at 1200 °C.

Figure 12 exhibits the SEM impact fractographs of crack healing zones after a 20% height reduction at different temperatures.

At 850 °C, the internal crack began to heal and the impact fracture presented the mixed fracture characterized by unhealed region and dimpled pattern. However, only a small fraction of the fracture presented the dimpled pattern. When the healing temperature was 950 °C, the remanent void coalescence mechanism prevailed in the dimpled region. The dimples were roughly equiaxed, as depicted in Figure 12a,b.

As showed in Figure 12c, when the healing temperature was 1050 °C, the fracture surface showed a river-like pattern cleavage that was surrounded by dimples of different sizes, both of them belonging to the fracture mechanism by the coalescence of the micro-void and quasi-cleavage mechanism. The internal cracks were fully healed at 1050 °C, which corresponded to the maximum percentage recovery.When the healing temperature was further heightened to 1150 °C and 1200 °C, the percentage recovery of the impact property descended gradually. The river pattern cleavage region was enlarged, which was in accordance with the evolution of grain coarsening, shown in Figure 12d,e.

**Figure 13:**
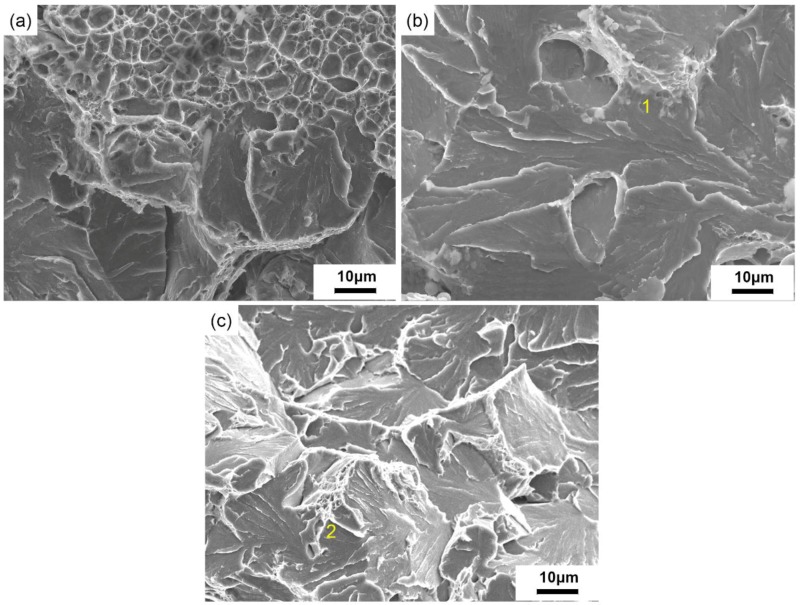
The SEM impact fracture of SA 508–3 under reduction ratios when the deformation temperature is 950 °C: (**a**) 10%, (**b**) 20%, and (**c**) 30%.

Figure 13 shows the SEM impact fracture of SA 508–3 steel with different reduction ratios; when the healing temperature was 950 °C, the internal crack was not completely healed under the condition of a 10% reduction ratio. The fracture surfaces were predominantly unhealed regions with the dimpled regions, as shown in Figure 13a.

When the reduction ratio reached 20% and 30%, the recovery percentage increased gradually, with similar fracture features. As seen in Figure 13b,c, the fracture surfaces were mainly river-like cleavage fractures. This rupture mechanism model involves a typical fracture surface morphology which consists of an array of cleavage facets or transgranular brittle fracture facets. Meanwhile, several dimples can be seen in region 1 and 2.

### 3.4. Fatigue Property Recovery

The standard fatigue cyclic life of the SA 508–3 material was nearly 24,000. The fatigue properties of 20% compressed SA 508–3 samples healed at 1050 °C for 10 h were tested, which had a good percentage recovery of the impact property. However, the tested specimen cycled for only 1065 times when fatigue failure occurred, even less than 10% of its original cyclic life.

**Figure 14:**
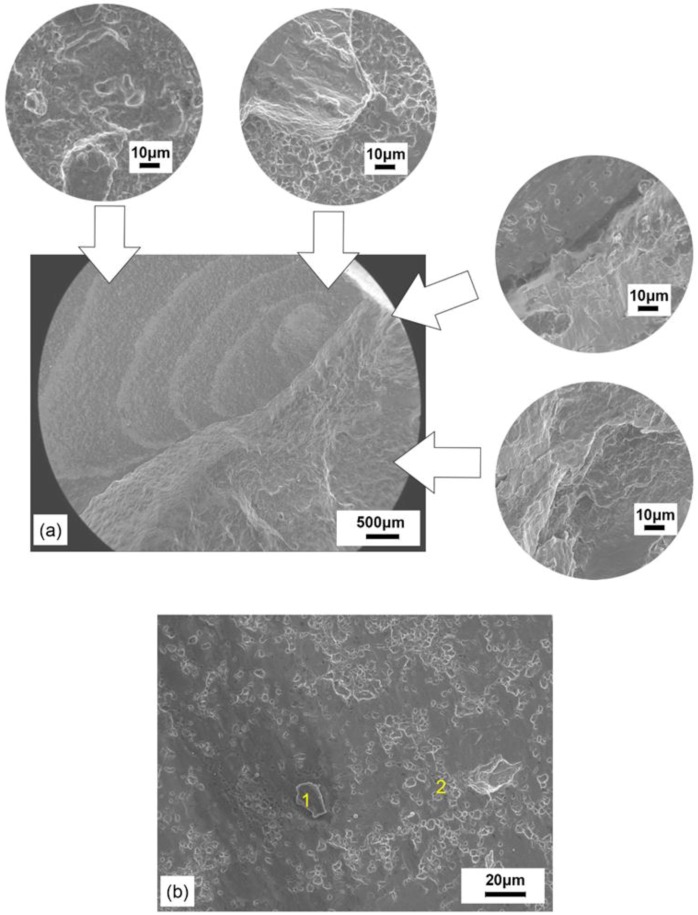
SEM fatigue fractographs of the SA 508–3 specimen healed at 1050 °C with a 20% reduction ratio: (**a**) the fatigue fracture morphology and (**b**) the ingredients of inclusion.

The fatigue fracture was located in the crack healing zone. The SEM images of fatigue fracture surface of SA 508–3 steel are shown in Figure 14. It should be noted that half of the crack healing zone presented characteristics similar to fatigue fracture, and half showed tensile fracture characteristics.

The quasi-fatigue fracture had the morphology of micro-void and unhealed regions, as shown in Figure 14a. Generally, the typical morphology of the fatigue fracture was identified by fatigue striation. However, no obvious fatigue striation or tear ridge could be observed in the quasi-fatigue fracture. Instead, micro-voids and inclusions were observed.

As shown in Figure 14b, inclusions (location 1) and micro-voids (location 2) could be observed on the quasi-fatigue surface. It was obtained by composition measurement that location 1 had a relatively high volume of chemical composition of O, which indicated the existence of nonmetallic inclusions, such as oxides.

As is well-known, the fatigue life would significantly reduce with the presence of nonmetallic inclusions. It was because the presence of inclusions, usually brittle particles, made reverse slip difficult on the slip plane during the loading and unloading process. Thus, brittle particles moved along the persistent slip bands. With subsequent cyclic loading, the microcrack initiated along the inclusion boundary. Meanwhile, the microcracks propagated with the remanent voids, which could not fully eliminate the attendant extension during the tension load in fatigue cycles. Then, they promoted fatigue failure.

From the fatigue fracture analysis, it can be concluded both that the inclusions assisted damage as well as the remnant internal voids were responsible for the reduction in the fatigue life of the SA 508–3 specimen. 

## 4. Discussion

The original microstructure of the investigated steel was characterized by martensite and lath bainite, as shown in Figure 3. When it was heated to the healing temperatures, which were above Ac3, a complete transformation of austenite in the matrix occurred.

Xin [22] has reported that, at the low healing temperature (<900 °C), the main internal crack healing mechanism was diffusion, the iron atoms migrated towards the prior austenite grain boundary along the preset crack, and then the crack width decreased. When the healing temperature further increased, a large number of ferrite grains aggregated near the crack healing zone.

In this study, when the healing temperature was 850 °C, the crack width decreased due to the atom migration that happened near the interface of the crack. After holding for 10 h, the grain growth led to a further reduction of the crack width.

When the temperature increased to 950 °C, the long cracks were segmented into small cracks with the appearance of recrystallized grains, and then, the small cracks transformed into spherical voids in the recrystallized grain. It was noted that the voids were observed inside the newly formed grains after the process of heat preservation.

When the healing temperature increased to 1050 °C, it is noteworthy that, after the hot compression, the two separated surfaces can be bonded partly with some renament voids. Also, the grain growth promoted the crack healing gradually with the increasing holding time.

When compressed at temperatures higher than 1150 °C, recrystallization occurs preferentially along the interface of crack and the initial recrystallization grains were difficult to cross the bonding interface due to interface incompatibility and the obstruction of the interfacial oxides, as Xu [23] studied. However, after holding at the healing temperature for 10 h, the initial recrystallization grains along both sides of the crack interface were able to grow up and the original crack became part of the newly formed grains boundary, as showed in Figure 5d,e. A complete interface healing could be achieved because of the grain boundary migration.

Meanwhile, in the study of SA 508–3 steel, after holding at the healing temperature for 10 h, the complete homogeneity of the microstructure in both the matrix and healing zone was achieved due to high-temperature diffusion. When the samples were cooling to the room temperature, the microstructure was only characterized by granular bainite. On the other hand, as shown in Figure 6, there was no significant differences in the fraction of small-angle boundaries or high-angle boundaries in the crack healing zone and the matrix. Therefore, it was assumed that the granular bainite was in equilibrium in this paper.

**Figure 15:**
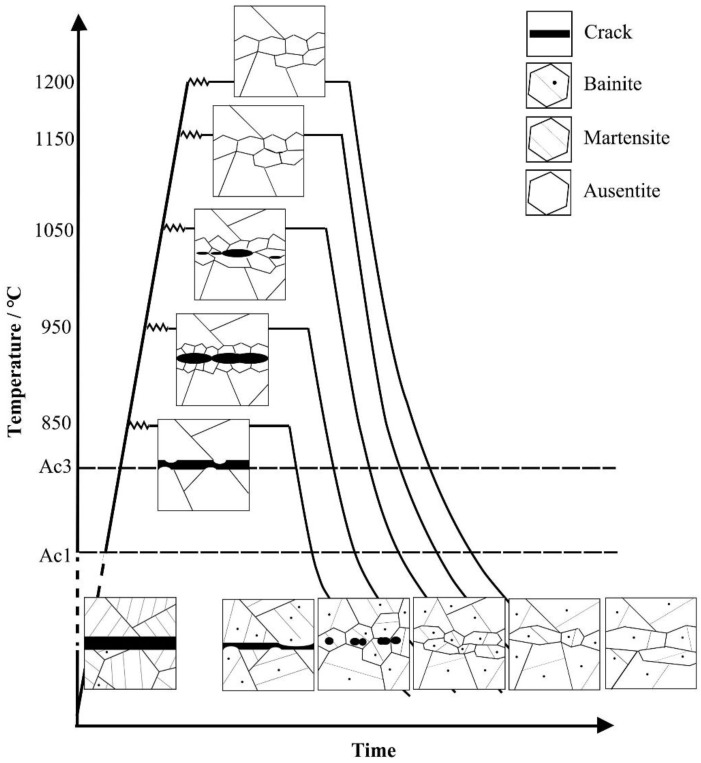
A schematic of the microstructural evolution of the crack zone in SA 508–3 steel.

In terms of the above discussions, the microstructure evolution in the healing process can be sketched in a schematic diagram as seen in Figure 15.

The interaction between microstructure and toughness is mainly dependent on two factors: the crack nucleus and the microstructure barriers that hinder crack propagation [7].

Internal crack healing was achieved by bringing atomic contact of the metallic free surface, and the remnant cracks and voids at the interface was the source of crack initiations. With the increase of healing temperature, the quantity and size of internal cracks decreased sharply, which improved the recovery degree of mechanical properties.

When the temperature increased to 1050 °C, an interface would not be observed at a higher healing temperature, which means the interface was completely bonded. It was observed that grain coarsening occurred after holding for 10 h.

The block boundaries of the granular bainite were the high-angle boundaries, and the fraction of small-angle boundaries within the blocks in the matrix was similar to those in the newly formed grains. When the grains grew up, the density of high-angle boundaries decreased relatively. Without the resistance of high-angle boundaries to impact the load, the stress concentration in large-size gains was more significant than that of small-size grains, causing great damage to the impact toughness.

Lan [24] pointed out that large MA constituents in a coarse bainite matrix are primary responsible for the low toughness because the interfacial decohesion between the MA constituents and the surrounding bainitic matrix stimulates the formation of the microcrack, which leads to cleavage fracture in the end. Meanwhile, as shown in Figure 5d,e, when the healing temperature increased, the size of the MA constituents increased.

## 5. Conclusions

Hot plastic deformation was applied to internal crack healing in SA 508–3. The effects of the experiment parameters on the microstructure evolution, as well as the relationship between the microstructure of the crack healing zone and mechanical properties were studied.

1. When the internal crack in SA 508–3 steel disappeared completely, the impact and low fatigue properties of the crack healing zones could only be partially achieved. The percentage recovery degrees increased with increasing healing temperature and reduction ratio in the temperature range of 950–1050 °C. When temperature was above 1150 °C, the impact properties began to deteriorate.
2. When the healing temperature increased from 850 °C to 1200 °C, the change of the morphology of the original crack experienced several phases: it segmented into short cracks, became spherical voids, became part of the newly formed grains boundary, and was completely replaced by grown grains.
3. When the crack was partially healed, the existence of remnant cracks and voids was the source of crack initiation. When the crack disappeared, the grain growth and large MA constituents led to the deterioration of the mechanical property.

## Figures and Tables

**Table 1:** The room-temperature impact properties of crack healing zones under different reduction ratios and healing temperatures.

Healing Temperature (°C)	Reduction Ratio (%)	Charpy Absorbed Energy (J)	Standard Deviation (J)
850	10	—	—
	20	6.34	0.398
	30	9.68	0.251
950	10	10.39	0.287
	20	15.98	0.611
	30	20.25	0.959
1050	10	18.19	0.719
	20	24.16	1.080
	30	27.52	0.976
1150	10	8.31	0.786
	20	12.04	1.050
	30	18.34	1.067
1200	10%	5.85	0.623
	20%	7.62	1.006
	30%	11.30	0.732

* The table listed the average values of four specimens at each data point.
